# Emergency Department Care for Children During the 2022 Viral Respiratory Illness Surge

**DOI:** 10.1001/jamanetworkopen.2023.46769

**Published:** 2023-12-07

**Authors:** Alexander T. Janke, Courtney W. Mangus, Christopher M. Fung, Neil Kamdar, Michelle L. Macy, Michele M. Nypaver, Keith E. Kocher

**Affiliations:** 1 National Clinician Scholars Program, Veterans Affairs Ann Arbor Healthcare System, Ann Arbor, Michigan; 2 Department of Emergency Medicine, University of Michigan, Ann Arbor; 3 Institute for Healthcare Policy and Innovation, University of Michigan, Ann Arbor; 4 Department of Pediatrics, University of Michigan, Ann Arbor; 5 Institute for Healthcare Policy and Innovation, University of Michigan, Ann Arbor; 6 Department of Pediatrics, Feinberg School of Medicine, Northwestern University, Chicago, Illinois; 7 Ann & Robert H. Lurie Children’s Hospital of Chicago, Chicago, Illinois; 8 Department of Learning Health Sciences, University of Michigan, Ann Arbor

## Abstract

**Importance:**

Pediatric readiness is essential for all emergency departments (EDs). Children’s experience of care may differ according to operational challenges in children’s hospitals, community hospitals, and rural EDs caused by recurring and sometimes unpredictable viral illness surges.

**Objective:**

To describe wait times, lengths of stay (LOS), and ED revisits across diverse EDs participating in a statewide quality collaborative during a surge in visits in 2022.

**Design, Setting, and Participants:**

This retrospective cohort study included 25 EDs from the Michigan Emergency Department Improvement Collaborative data registry from January 1, 2021, through December 31, 2022. Pediatric (patient age <18 years) encounters for viral and respiratory conditions were analyzed, comparing wait times, LOS, and ED revisit rates for children’s hospital, urban pediatric high-volume (≥10% of overall visits), urban pediatric low-volume (<10% of overall visits), and rural EDs.

**Exposures:**

Surge in ED visit volumes for children with viral and respiratory illnesses from September 1 through December 31, 2022.

**Main Outcomes and Measures:**

Prolonged ED visit wait times (arrival to clinician assigned, >4 hours), prolonged LOS (arrival to departure, >12 hours), and ED revisit rate (ED discharge and return within 72 hours).

**Results:**

A total of 2 761 361 ED visits across 25 EDs in 2021 and 2022 were included. From September 1 to December 31, 2022, there were 301 688 pediatric visits for viral and respiratory illness, an increase of 71.8% over the 4 preceding months and 15.7% over the same period in 2021. At children’s hospitals during the surge, 8.0% of visits had prolonged wait times longer than 4 hours, 8.6% had prolonged LOS longer than 12 hours, and 42 revisits occurred per 1000 ED visits. Prolonged wait times were rare among other sites. However, prolonged LOS affected 425 visits (2.2%) in urban high-pediatric volume EDs, 133 (2.6%) in urban pediatric low-volume EDs, and 176 (3.1%) in rural EDs. High visit volumes were associated with increased ED revisits across sites.

**Conclusions and Relevance:**

In this cohort study of more than 2.7 million ED visits, a pediatric viral illness surge was associated with different pediatric acute care across EDs in the state. Clinical management pathways and quality improvement efforts may more effectively mitigate dangerous clinical conditions with strong collaborative relationships across EDs and setting of care.

## Key Points

**Question:**

Was a surge in viral and respiratory cases differentially associated with emergency department (ED) care at children’s hospitals in a statewide quality collaborative?

**Findings:**

In this cohort study of more than 2.7 million ED visits, prolonged wait times and lengths of stay were common at children’s hospitals. Greater visit volumes were associated with increased likelihood of ED revisits across hospitals.

**Meaning:**

Periods of illness surge have differential associations with ED care for children by setting, highlighting the importance of coordinating pediatric readiness and surge preparedness across diverse EDs.

## Teaser

This cohort study describes wait times, lengths of stay, and revisits for pediatric patients with acute viral and respiratory conditions presenting at Michigan emergency departments (EDs) during a surge of visits in 2022.

## Introduction

Emergency department (ED) operations require that staffing, facility, and equipment availability are balanced with patient needs to maintain high-quality care, especially during periods of system stress. An important component of operational planning is pediatric readiness. Where pediatric expertise and equipment are promptly available, mortality for critically ill children is reduced.^1^ Respiratory conditions are among the most common potentially serious illnesses for which children present for emergency care.^2^ Periods of respiratory illness surge may weaken standard ED care processes, especially for more vulnerable patients, those with complex chronic conditions,^3^ or those in community or rural EDs.

Early in the COVID-19 pandemic, there was a precipitous decrease in pediatric ED visits for respiratory illness to less than 40% of baseline,^4^ but this was short-lived. More recently, beginning in September 2022, pediatric ED visits in the US for respiratory concerns surged with a wave of COVID-19, influenza, and respiratory syncytial virus (RSV) infections.^5,6,7^ In research among adults, health system strain during periods of increased demand for acute care during the COVID-19 pandemic has been shown to contribute to overall population-level mortality.^8,9^ More generally, an extensive research literature has demonstrated the harmful effects of emergency department (ED) “crowding,” where available staff and space are not on hand to meet patient care demands.^10,11^ Access to emergency care and safe clinical operations may be compromised when wait times and lengths of stay (LOS) are severely prolonged.

To date, little research has described how health system strain affects acute care for children with viral and respiratory conditions across diverse hospital-based EDs. Three accessible and patient-relevant operations metrics for ED care are ED wait times, LOS, and ED revisits following a discharge.^12^ These measures represent the timeliness of care, the availability of ED and hospital-based resources, and, in the case of ED revisits, the potential accessibility of outpatient follow-up care.^13^ The goal of this investigation was to describe wait times, LOS, and ED revisits for pediatric patients with acute viral and respiratory conditions across a wide range of EDs participating in a statewide quality improvement collaborative data registry, with a focus on a period of surge in pediatric illness and ED patient volumes.

## Methods

### Setting and Participants

This was a retrospective cohort study of patients younger than 18 years presenting to EDs participating in the Michigan Emergency Department Improvement Collaborative^14,15^ (MEDIC) from January 1, 2021, to December 31, 2022. The study was classified as exempt by the Institutional Review Board at the University of Michigan, Ann Arbor. This study adhered to the Strengthening the Reporting of Observational Studies in Epidemiology (STROBE) reporting guideline.

The MEDIC data registry includes electronic health record–extracted data elements for all ED visits among participating sites. We identified September 1 to December 31, 2022, as the time frame for focused analysis of the surge based on the rapid increase in pediatric ED visits in that period. We stratified the ED site types as children’s hospitals, urban EDs with high pediatric volume (pediatric visits were ≥10% of the total visits), urban EDs with low pediatric volume (pediatric visits were <10% of the total visits), and rural community EDs, based on the premise that ED operations and capacity to respond to pediatric surge are likely to vary significantly among these groups. Urban-rural designations were defined per standardized Rural-Urban Commuting Area codes as implemented in prior US research on locations of EDs.^16^

We included ED visits based on review of all chief problems and diagnosis codes identified as likely attributable to the viral and respiratory illness surge in 3 steps. First, we included ED visits with a chief complaint for fever and/or respiratory signs and symptoms (eg, cough, rhinorrhea, shortness of breath). Second, we included visits with an *International Classification of Diseases, Tenth Revision, Clinical Modification* (*ICD-10-CM*), diagnosis code (primary diagnosis or secondary diagnoses) for an infectious respiratory condition using the Healthcare Cost and Utilization Project Clinical Classification Software Revised.^17^ These codes are broadly inclusive of common pediatric respiratory conditions such as asthma, croup, bronchiolitis, pneumonia, and respiratory failure. Third, we included ED visits with any *ICD-10-CM* code consistent with an acute viral syndrome, including syndromic codes (such as R06.02 [shortness of breath] and R50.9 [fever, unspecified]), as well as those specific to a particular viral pathogen (such as Z20.822 [contact with and suspected exposure to COVID-19] and J12.2 [*parainfluenzavirus* pneumonia]). eTables 1 and 2 in Supplement 1 detail the most common chief problems and *ICD-10-CM* codes used to define the cohort of pediatric respiratory and viral ED visits for analysis.

### Outcomes and Measures

Outcomes included median (IQR) ED wait times and LOS reported in hours, count and proportion of ED visits with prolonged wait times (>4 hours) and LOS (>12 hours), as well as rate of ED discharge with subsequent return within 72 hours (per 1000 ED visits). Wait time was defined as the first available time stamp for the encounter (arrival/triage) to the time a clinician was assigned; LOS was defined as the first available time stamp for the encounter to ED departure time stamp. We chose to dichotomize outcomes for prolonged wait times and LOS because of observed small changes in medians over time and because adverse consequences for patients are likely to be concentrated among those at these extremes (eFigures 1 and 2 in Supplement 1). The 4- and 12-hour thresholds were specified after review of the distribution of these outcomes, to target relatively face-valid time frames for problematic waits and LOS. For a small number of visits at several sites, patients are placed in ED observation care, as described in the Results section. The MEDIC data registry does not allow us to distinguish the time when ED observation care is initiated. This means that for the small number of cases where observation care took place, LOS is total time in ED inclusive of initial ED care and observation care.

We report sociodemographic characteristics, acuity (emergency severity index [ESI] score, ranging from 1 to 5, with higher scores indicating lower acuity), diagnoses, and disposition among sample ED visits. We included demographic variables for age, sex, and race and ethnicity. Age was categorized as younger than 4 weeks, 4 weeks to 3 months, 4 to 23 months, 2 to 4 years, 5 to 11 years, and 12 to 17 years based on existing guidelines for the clinical care of children with conditions such as fever and asthma.^18,19^ Data for patient sex were drawn from electronic health records across sites, and information clarifying this variable vs patient gender or gender identity were not readily available. Where available, race and ethnicity were categorized in accordance with best practices in health services research.^20^ However, race and ethnicity data collection are both limited and with likely heterogeneous reporting mechanisms across participating EDs and in the MEDIC data registry. Race data were categorized as American Indian or Alaska Native, Asian, Black or African American, Native Hawaiian or Other Pacific Islander, White, more than 1 race, and other race or ethnicity. Ethnicity data were categorized as Hispanic or Latino and non-Hispanic or non-Latino.

### Statistical Analysis

To place the visit volumes during the surge from September 1 to December 31, 2022, in the context of recent trends in ED visit rates, weekly visit counts were plotted from January 1, 2021, through December 31, 2022, and we compared total visits during the chosen surge period (September 1 to December 31, 2022) with those of the preceding 4-month period (May 1 to August 31, 2022) as well as the same months in the preceding year (September 1 to December 31, 2021).

To examine the association between shifting pediatric viral and respiratory visit volumes and our outcomes, we created a *z* score for daily visits for these patients at each site across the entire 2-year study period. We report odds ratios (ORs) for ED visits’ likelihood of resulting in prolonged wait times (>4 hours), prolonged LOS (>12 hours), and ED revisit after discharge (within 72 hours) across ED types according to each site’s daily pediatric viral and respiratory visits *z* score (<1 SD above the 2-year study period mean visit volume, labeled moderate or low volume; 1-2 SDs above the mean, high volume; and >2 SDs above the mean, highest volume).

We then focused on the identified surge period (September 1 to December 31, 2022), to address how wait times, LOS, and revisits varied across different sites during the surge. We tabulated monthly outcomes for each of the 4 months in the period and compared odds of outcomes across site types. Urban community EDs with 10% or greater pediatric volume were used as the reference category for those comparisons, given that these EDs likely represent the plurality of ED visits for children nationwide.^21^ Given the likely significant case-mix variation among EDs, ORs are reported as unadjusted as well as adjusted for age, sex, race and ethnicity, ESI, and presence of complex chronic conditions (according to the Feudtner classification system^22^).

Data cleaning and tabulations were performed in R, version 4.1.2 (R Project for Statistical Computing). Statistical modeling was performed in Stata, version 18 (StataCorp LLC). Two-sided *P* < .05 indicated statistical significance.

## Results

A total of 2 761 361 total adult and pediatric ED visits across 25 EDs in 2021 and 2022 were retrieved. The sample included 3 pediatric referral hospitals, 9 urban sites with high pediatric volume (≥10%), 7 urban sites with low pediatric volume (<10%), and 6 rural community EDs (eTable 3 in Supplement 1). There were 301 688 total pediatric viral and respiratory visits (inclusive of 53.1% of all pediatric visits and 10.9% of total ED visits in the study). The most common diagnoses in the cohort (eTable 2 in Supplement 1) were for signs and symptoms of upper respiratory tract infection (40 181 [13.3%]), fever, unspecified (24 011 [8.0%]), and COVID-19 (13 985 [4.6%]). eTables 4 and 5 in Supplement 1 list available sociodemographic and visit characteristics across ED types (52.4% male and 47.6% female patients; most patients [28.3%] aged 4-23 months). In terms of race, 0.03% of patients were American Indian or Alaska Native, 1.0% were Asian, 30.9% were Black, 0.1% were Native Hawaiian or Other Pacific Islander, 40.4% were White, 2.4% were more than 1 race, and 19.7% were of other race. In terms of ethnicity, 9.7% were Hispanic or Latino and 90.0% were non-Hispanic or non-Latino. Children’s hospitals cared for a larger proportion of Black patients, patients with higher-acuity and more complex chronic conditions, and patients with higher admission rates. Conversely, rural sites treated more White patients with lower-acuity conditions and higher discharge rates. Beginning in September 2022, visits for viral and respiratory illnesses began to rise and peaked from November to December 2022. During that period and across ED types, there were a total of 74 282 such visits, an increase of 71.8% compared with the 4 preceding months and 15.7% more than during the same period in 2021 (Figure). The surge period from September to December 2022 comprised all the highest-volume days (>2 SDs above the 2-year study period mean) for visits for pediatric viral and respiratory illnesses.

**Figure.**
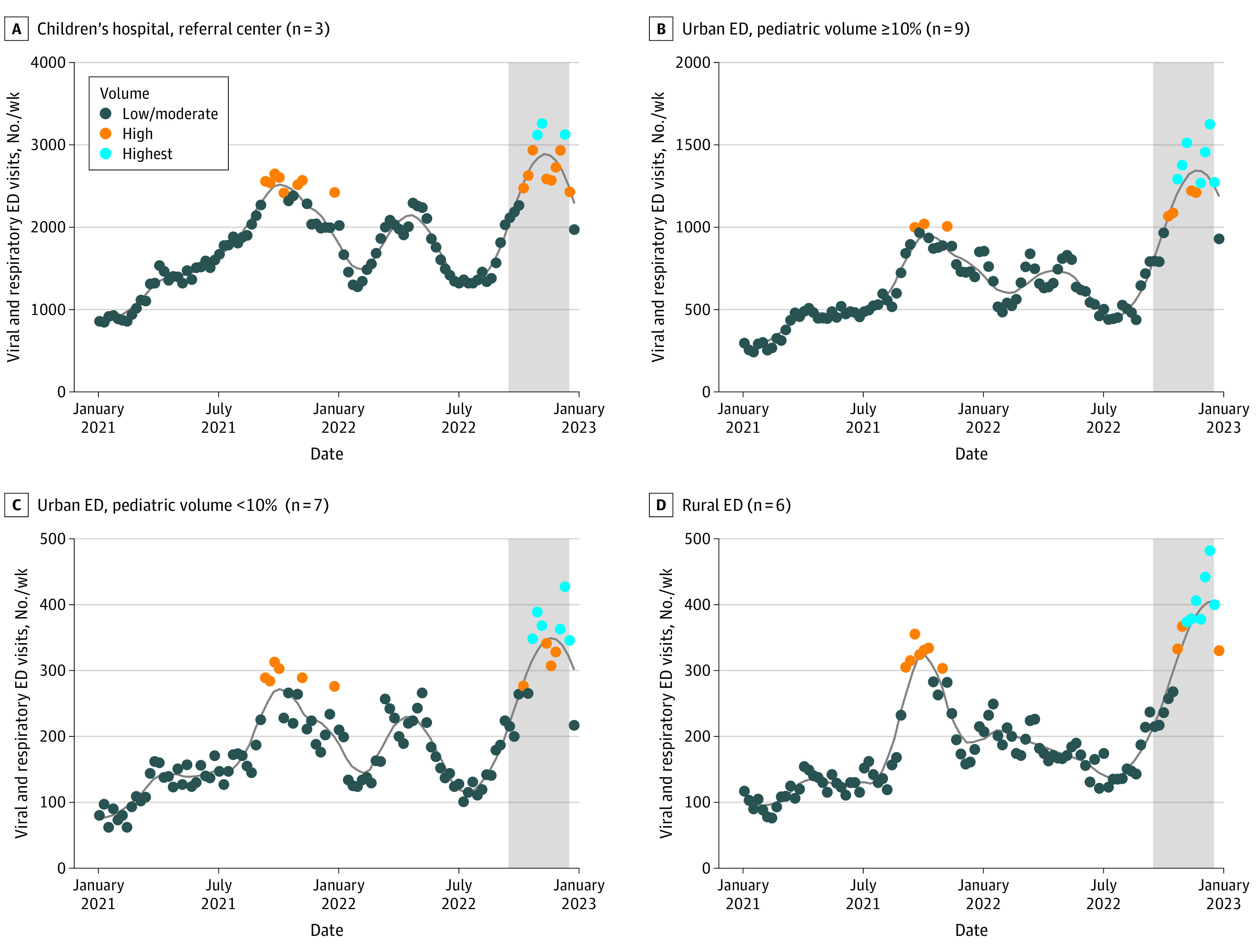
Trends in Weekly Pediatric Viral and Respiratory Visits at 25 Michigan Emergency Departments (EDs) Sample includes 25 EDs from the Michigan Emergency Department Improvement Collaborative (MEDIC). Weekly visit counts are depicted for 2021 and 2022 and indicate low- and moderate-volume (*z* score, <1), high-volume (*z* score, 1-2), and highest-volume (*z* score, >2) EDs. Michigan experienced a significant surge in pediatric respiratory cases related to influenza, COVID-19, and respiratory syncytial virus infections beginning in September 2022 (shaded). Smoothed curves are generated using locally weighted scatterplot smoothing (span, 0.32) with 95% CI regions using previously described methods.

Daily visit volumes were variably associated with wait times, LOS, and ED revisits across site types (Table 1). For example, compared with moderate- or low-volume days at children’s hospitals, highest-volume days were associated with increased wait times longer than 4 hours (OR, 4.09 [95% CI, 3.79-4.42]), LOS longer than 12 hours (OR, 1.37 [95% CI, 1.26-1.48]), and ED revisits (OR, 1.40 [95% CI, 1.28-1.53]). In contrast, daily pediatric viral and respiratory visit volumes were not consistently associated with prolonged wait times at other sites. Rural EDs, though, had greater LOS on high- (OR, 1.47 [95% CI, 1.15-1.88]) and highest-volume days (OR, 2.06 [95% CI, 1.59-2.64]), despite a lack of association on these measures for other community EDs. Prolonged LOS was generally associated with higher-acuity visits, and more than 200 ED visits in the sample resulting in transfer had an LOS greater than 12 hours (eTable 6 in Supplement 1). Across site types, highest-volume days were associated with greater ED revisits within 72 hours as compared with low- or moderate-volume days, though ORs were not statistically significant in the case of urban, low pediatric volume sites. A minority of ED revisits (from as low as 9.7% at rural sites to as high as 26.2% at children’s hospitals) resulted in hospitalization (eTable 7 in Supplement 1).

**Table 1.** Odds Ratios for Prolonged Wait Times, LOS, and 72-Hour Revisit Over Time, January 1, 2021, to December 31, 2022

Day’s visit volume	*z* Score^a^	OR (95% CI)^b^		
		Wait time >4 h	LOS >12 h	ED revisit within 72 h
**Children’s hospital EDs (n = 3)**				
Moderate or low	<1	1 [Reference]	1 [Reference]	1 [Reference]
High	1-2	3.61 (3.42-3.81)	1.55 (1.48-1.62)	1.08 (1.01-1.14)
Highest	>2	4.09 (3.79-4.42)	1.37 (1.26-1.48)	1.40 (1.28-1.53)
**Urban EDs, pediatric volume ≥10%**				
Moderate or low	<1	1 [Reference]	1 [Reference]	1 [Reference]
High	1-2	1.14 (0.80-1.59)	0.93 (0.81-1.06)	1.27 (1.14-1.41)
Highest	>2	1.03 (0.66-1.53)	0.95 (0.81-1.11)	1.57 (1.40-1.75)
**Urban EDs, pediatric volume <10%**				
Moderate or low	<1	1 [Reference]	1 [Reference]	1 [Reference]
High	1-2	1.33 (1.02-1.72)	1.02 (0.82-1.25)	1.13 (0.85-1.47)
Highest	>2	1.09 (0.77-1.51)	0.81 (0.61-1.06)	1.38 (1.02-1.84)
**Rural EDs**				
Moderate or low	<1	1 [Reference]	1 [Reference]	1 [Reference]
High	1-2	1.49 (1.07-2.05)	1.47 (1.15-1.88)	1.22 (1.03-1.44)
Highest	>2	1.12 (0.72-1.67)	2.06 (1.59-2.64)	1.32 (1.08-1.59)

Abbreviations: ED, emergency department; LOS, lengths of stay; OR, odds ratio.

^a^ Calculated for daily pediatric viral and respiratory arrivals with respect to the mean and SD for daily pediatric viral and respiratory arrivals across the full 2-year period and across site types.

^b^ Reported within site types for ED visit likelihood of prolonged wait times (>4 hours), prolonged LOS (>12 hours), and ED revisit (within 72 hours) according to visit volume on day of arrival.

Daily visit volumes rose and peaked in the identified surge period from September 1 to December 31, 2022. There was considerable variation in wait times, LOS, and ED revisit rates across site types during that period (Table 2). While median wait times were generally less than 1 hour across EDs, wait times greater than 4 hours were common at children’s hospitals (occurring in 3504 visits [8.0%] during the surge period) and rare at other sites. An LOS longer than 12 hours was also more common at children’s hospitals (occurring in 3789 visits [8.6%]) compared with urban sites with low pediatric volume (133 [2.6%]), urban sites with high pediatric volume (425 [2.2%]), and rural EDs (176 [3.1%]). In addition, at their peak volume in November, 84 of 1658 rural ED visits (5.1%) exhibited LOS longer than 12 hours. After adjusting for age, sex, race and ethnicity, ESI acuity, primary diagnosis, and complex chronic conditions (adjusted OR [AOR]), the odds of wait times longer than 4 hours and LOS longer than 12 hours for ED visits were greater during the surge at children’s hospitals (AORs, 51.43 [95% CI, 37.76-70.04] and 3.82 [95% CI, 3.44-4.26], respectively), urban sites with low pediatric volume (AORs, 10.24 [95% CI, 7.13-14.71] and 1.02 [95% CI, 0.82-1.25], respectively), and rural EDs (AORs, 5.17 [95% CI, 3.42-7.81] and 1.85 [95% CI, 1.53-2.22], respectively) compared with urban sites with high pediatric volume (Table 3). Of the 4523 total visits with LOS longer than 12 hours during the identified surge period (Table 2), 78 (1.7%) at 5 EDs in the sample included ED observation care. Emergency department revisit rates by site type and month varied from 16 to 66 per 1000 visits (eg, 42 per 1000 visits at children’s hospitals) and were highest at rural sites.

**Table 2.** Pediatric Viral and Respiratory Visits During the Surge, September to December 2022

Month	Total No. of visits	Wait times		LOS		Revisits
		Median (IQR), h	>4 h, No. (%)	Median (IQR), h	>12 h, No. (%)	Within 72 h, No./1000
**Children’s hospital EDs (n = 3)**						
September	8545	0.72 (0.27-1.78)	613 (7.2)	3.50 (1.88-6.46)	789 (9.2)	36
October	11 429	1.03 (0.39-2.38)	1100 (9.6)	3.47 (1.90-6.25)	1059 (9.3)	46
November	12 377	0.96 (0.42-2.13)	1091 (8.8)	3.42 (1.93-6.15)	1170 (9.5)	48
December	11 506	0.73 (0.30-1.69)	700 (6.1)	3.05 (1.76-5.45)	771 (6.7)	36
**Urban EDs, pediatric volume ≥10% (n = 9)**						
September	3277	0.35 (0.18-0.72)	<10	2.60 (1.73-3.97)	77 (2.3)	37
October	4873	0.40 (0.21-0.80)	<10	2.55 (1.65-3.88)	84 (1.7)	47
November	5732	0.41 (0.20-0.82)	<10	2.52 (1.65-3.83)	178 (3.1)	58
December	5773	0.40 (0.20-0.87)	24 (0.4)	2.42 (1.67-3.62)	86 (1.5)	36
**Urban EDs, pediatric volume <10% (n = 7)**						
September	879	0.50 (0.23-1.11)	15 (1.7)	2.98 (1.82-4.62)	24 (2.7)	18
October	1279	0.57 (0.28-1.32)	20 (1.6)	3.17 (1.93-4.58)	33 (2.6)	23
November	1494	0.62 (0.27-1.33)	31 (2.1)	3.13 (2.05-4.88)	45 (3.0)	31
December	1493	0.66 (0.27-1.52)	41 (2.7)	3.07 (1.87-4.60)	31 (2.1)	16
**Rural EDs (n = 6)**						
September	928	0.35 (0.13-0.92)	12 (1.3)	2.08 (1.25-3.40)	17 (1.8)	31
October	1240	0.30 (0.11-0.76)	12 (1.0)	1.98 (1.22-3.00)	17 (1.4)	49
November	1658	0.30 (0.12-0.80)	14 (0.8)	1.99 (1.23-3.19)	84 (5.1)	66
December	1799	0.31 (0.11-1.01)	13 (0.7)	2.05 (1.38-3.10)	58 (3.2)	39

Abbreviations: ED, emergency department; LOS, lengths of stay.

**Table 3.** Odds Ratios for Prolonged Wait Times, LOS, and 72-Hour Revisit Across Site Types During the Surge

Type of ED	No. of visits	OR (95% CI)	AOR (95% CI)^a^
**Wait times >4 h^b^**			
Children’s hospital (n = 3)	3504 (8.0)	41.78 (30.69-56.86)	51.43 (37.76-70.04)
Urban, pediatric volume ≥10% (n = 9)	42 (0.2)	1 [Reference]	1 [Reference]
Urban, pediatric volume <10% (n = 7)	107 (2.1)	10.90 (7.59-15.64)	10.24 (7.13-14.71)
Rural (n = 6)	51 (0.9)	4.33 (2.86-6.54)	5.17 (3.42-7.81)
**LOS >12 h^b^**			
Children’s hospital (n = 3)	3789 (8.6)	4.22 (3.81-4.69)	3.82 (3.44-4.26)
Urban, pediatric volume ≥10% (n = 9)	425 (2.2)	1 [Reference]	1 [Reference]
Urban, pediatric volume <10% (n = 7)	133 (2.6)	1.16 (0.95-1.42)	1.02 (0.82-1.25)
Rural (n = 6)	176 (3.1)	1.47 (1.22-1.75)	1.85 (1.53-2.22)
**ED revisit <72 h^c^**			
Children’s hospital (n = 3)	1837 (42)	0.91 (0.84-0.99)	0.90 (0.83-0.98)
Urban, pediatric volume ≥10% (n = 9)	887 (45)	1 [Reference]	1 [Reference]
Urban, pediatric volume <10% (n = 7)	116 (23)	0.49 (0.40-0.59)	0.54 (0.44-0.65)
Rural (n = 6)	271 (48)	1.08 (0.93-1.24)	1.14 (0.99-1.31)

Abbreviations: AOR, adjusted odds ratio; ED, emergency department; LOS, lengths of stay; OR, odds ratio.

^a^ Adjusted for age, sex, race, ethnicity, emergency severity index acuity, primary diagnosis code, and history of or reporting of complex chronic conditions according to the Feudtner classification. The 95% CIs are calculated with adjustment for clustering at the site level.

^b^ Expressed as No. (%) of visits.

^c^ Expressed as No. (rate per 1000).

## Discussion

While increases in pediatric volume were universal across this diverse group of 25 EDs participating in MEDIC beginning September 1, 2022, the association of high visit volumes with prolonged wait times, prolonged LOS, and ED revisits were heterogeneous. Children’s hospitals exhibited stronger associations between visit volumes and these measures and with greater overall burden of operational strain. Emergency department visits during the identified surge period were 53 times more likely to have prolonged wait times greater than 4 hours at children’s hospitals compared with EDs with high pediatric volume. Case-mix adjustments available did not mitigate these differences between types of EDs, suggesting that otherwise similar patients can expect longer waits at children’s hospitals than at other EDs. Because visits for pediatric viral and respiratory illness comprise much of their overall care, children’s hospitals may be especially susceptible to viral illness surges among children, while ED operations at other sites may not be as sensitive to shifting pediatric care demands. Insofar as children’s hospitals are responsible for a growing proportion of inpatient care in the state, including accepting transfers from outlying sites, this may explain the bottleneck affecting wait times and LOS at those sites. At the same time pediatric volumes are growing, other sites may be experiencing decline or stable ED volumes for adult patients, permitting some flexibility to manage a surge in pediatric ED visits. In addition, ED observation care does not appear to significantly mitigate the finding of prolonged LOS, involved in only 1.7% of the ED visits with LOS longer than 12 hours during the identified surge period.

The mission of the MEDIC project is to improve ED care quality through a learning health system model.^23^ MEDIC partners with EDs within the network to support quality improvement through techniques such as audit and feedback measurement and reporting, support of evidence translation to practice change, and knowledge generation and dissemination. At the height of the 2022 respiratory viral surge period and as part of the All in for Kids campaign, the MEDIC coordinating center organized a virtual town hall with representatives from across the state to share operational challenges and identify approaches to mitigate them.^24^ Participants described shortages of beds and nursing expertise, insufficient pediatric readiness, and suboptimal coordination between small EDs and transfer centers^25^ as specific areas in need of support.

The American Academy of Pediatrics recently released a technical report in response to unprecedented pediatric patient volumes in late 2022, outlining steps that EDs can take to mitigate the deleterious effects of ED crowding.^26^ The report cites the need for standardized clinical management pathways, quality measurement development, and financial incentives such as pay-for-performance to ensure that children in children’s hospitals and community EDs alike receive timely and high-quality care. We observe that children’s hospitals are much more likely to exhibit large proportions of patients with prolonged wait times and LOS, which may contribute to dangerous conditions across the state if critically ill children requiring transfer at other sites are also affected. Concerningly, children in other EDs, especially in rural locations, were also affected by prolonged LOS in excess of 12 hours, and this may partially represent a referral network–wide capacity constraint. In addition, highest-volume days for ED visits were consistently associated with greater risk of return visits, which might reflect difficulty accessing outpatient follow-up care during surge periods, a higher threshold to admit patients when bed availability is limited, or lower-quality care in the ED setting. An essential feature of future pediatric readiness and surge preparedness efforts should be collaborative relationships across children’s hospitals, community EDs, rural sites, and outpatient pediatrics practices, as this will enhance the effectiveness of clinical management pathways and quality measures cited in the technical report on crowding.

The American Academy of Pediatrics report also outlines the influence of sociodemographic and community factors on both where crowding occurs and whom it adversely affects. Existing literature^27^ has demonstrated differences in wait times nationwide by race and ethnicity prior to the COVID-19 pandemic. Though limited by the absence of complete and high-quality data on race and ethnicity, we find that larger children’s hospital EDs are most adversely associated with crowding. That sites with greater strain may also care for a greater proportion of patients from racial and ethnic minority communities may be a feature of structural racism. That said, a nuanced interpretation of differences between sites must address both the contextual as well as compositional effects.^28^ Contextual effects, such as operational conditions, staffing and space availability, and underlying payer mix, may represent health policy and financing mechanisms requiring reform. Compositional effects, such as differences in underlying medical complexity of patients or severity of illness among patients presenting to children’s hospitals as opposed to community EDs, may represent more upstream drivers of poor health status. Importantly, crowded conditions may also contribute directly to clinician biases and decision-making that exacerbate disparities in care.^29,30^ Notably, our case-mix adjustments do not explain differences in rates of prolonged wait times, LOS, and ED revisits between children’s hospitals and other sites. Attention to differential effects of crowding across and within EDs is essential to mitigate harms during future periods of system strain.

### Limitations

This study has several important limitations. First, this was not a comprehensive assessment of all EDs in the State of Michigan, so our results may not be generalizable. A more detailed description of sites’ individual capacities, including pediatric readiness score or drive time to the closest pediatric intensive care unit, is not yet available in the MEDIC data registry. That said, we provide some context for the sites we assessed, including urban-rural designation, pediatric volume, hospitalization, and pediatric intensive care unit availability (eTable 3 in Supplement 1). Further, the MEDIC registry includes all of the dedicated stand-alone children’s hospitals in Michigan in addition to other EDs providing care to children in urban and rural areas. Second, differences in wait times, LOS, and revisits may reflect differences in patient case mix between EDs that we were not able to explore. Prolonged wait times and LOS are rare, especially at nonpediatric referral hospital sites, limiting our ability to adjust for differences in age, acuity, and chief complaints. For 1.7% of ED visits with prolonged LOS longer than 12 hours, ED observation care was documented. Thus, for those visits, prolonged LOS does not necessarily reflect problematic or strained care processes. Sites vary considerably in baseline operational characteristics, and our results should not be construed to imply that the surge explains baseline differences in prolonged wait times and LOS across site types. Third, while we attempted to isolate a cohort of acute viral and respiratory presentations, some patients may have presented for other reasons that resulted in prolonged wait times, prolonged LOS, or risk of ED revisit unrelated to the operational state of the ED (eg, mental health visits^31^). Fourth, while wait times, LOS, and revisits are important for patient experience and may be useful proxies for potentially dangerous clinical conditions, our work lacks other specific measures of health system strain, including total hospital occupancy or outcome measures such as mortality (rare in pediatric populations). Future work to follow up a cohort of pediatric patients over time, especially throughout their hospital stays for those requiring admission, will be essential.

Our findings notwithstanding, there is a paucity of data available to describe the differential effects of system strain across hospitals. Future pandemics, epidemics, and other disasters are likely to precipitate similar surges in acute care demands, and existing literature has clearly demonstrated the harms of ED crowding.^11^ Finally, race and ethnicity data in the MEDIC data registry are collected according to local practices without standardization across the network and with high rates of missingness. This limited our ability to address disparities for racial and ethnic minority groups within or across EDs. Nevertheless, our finding that different hospitals experience differential associations with prolonged wait times and LOS are consistent with ED crowding as a potential lever driving health care disparities.^32^

## Conclusions

In this cohort study of more than 2.7 million ED visits in Michigan, a pediatric viral illness surge was associated with different pediatric acute care across EDs in the state. Associations between visit volumes and the incidence of prolonged wait times, prolonged LOS, and ED revisits were heterogeneous. Future surges in acute care demand may have similarly differential impacts. Clinical management pathways and quality improvement efforts are more likely to effectively mitigate dangerous clinical conditions with strong collaborative relationships across different EDs and crossing settings of care. Policy makers and health system leaders should pursue a financial and operational model of pediatric acute care that rewards both pediatric readiness and surge preparedness to ensure that all children receive the best possible care.

## Notes

Supplement 1. **eTable 1.** Most Common Chief Complaints Defining Sample Cohort **eTable 2.** Most Common Diagnosis Codes Defining Sample Cohort **eTable 3.** Sample Emergency Department Sites from the Michigan Emergency Department Improvement Collaborative **eTable 4.** Sample Sociodemographic Characteristics of Pediatric Viral and Respiratory Visits **eTable 5.** Sample Pediatric Viral Respiratory Visit Characteristics **eTable 6.** High Acuity and Transfer Visits for Lengths of Stay >12 Hours During the Surge Period **eTable 7.** High-Acuity and Hospitalizations for ED Revisits During the Surge Period **eFigure 1**. Distribution of Wait Times Across Site Types for Pediatric Viral and Respiratory Visits, January 1, 2021, to December 31, 2022 **eFigure 2**. Distribution of Lengths of Stay Across Site Types for Pediatric Viral and Respiratory Visits, January 1, 2021, to December 31, 2022

Supplement 2.
Data Sharing Statement
