# Supplementary material for: Emergency Department Care for Children During the 2022 Viral Respiratory Illness Surge
Source: JAMA Netw Open. 2023 Dec 7;6(12):e2346769. doi: 10.1001/jamanetworkopen.2023.46769 (PMC10704279; doi:10.1001/jamanetworkopen.2023.46769)
Supplement: Supplement 1. — eTable 1. Most Common Chief Complaints Defining Sample Cohort eTable 2. Most Common Diagnosis Codes Defining Sample Cohort eTable 3. Sample Emergency Department Sites from the Michigan Emergency Department Improvement Collaborative eTable 4. Sample Sociodemographic Characteristics of Pediatric Viral and Respiratory Visits eTable 5. Sample Pediatric Viral Respiratory Visit Characteristics eTable 6. High Acuity and Transfer Visits for Lengths of Stay >12 Hours During the Surge Period eTable 7. High-Acuity and Hospitalizations for ED Revisits During the Surge Period eFigure 1. Distribution of Wait Times Across Site Types for Pediatric Viral and Respiratory Visits, January 1, 2021, to December 31, 2022 eFigure 2. Distribution of Lengths of Stay Across Site Types for Pediatric Viral and Respiratory Visits, January 1, 2021, to December 31, 2022 [file jamanetwopen-e2346769-s001.pdf]

Supplemental Online Content

Janke AT, Mangus CW, Fung CM, et al. Emergency department care for children during the 2022 viral respiratory illness surge. *JAMA Netw Open.* 2023;6(12):e2346769. doi:10.1001/jamanetworkopen.2023.46769

eTable 1. Most Common Chief Complaints Defining Sample Cohort

eTable 2. Most Common Diagnosis Codes Defining Sample Cohort

eTable 3. Sample Emergency Department Sites from the Michigan Emergency Department Improvement Collaborative

eTable 4. Sample Sociodemographic Characteristics of Pediatric Viral and Respiratory Visits

eTable 5. Sample Pediatric Viral Respiratory Visit Characteristics

eTable 6. High Acuity and Transfer Visits for Lengths of Stay >12 Hours During the Surge Period

eTable 7. High-Acuity and Hospitalizations for ED Revisits During the Surge Period

eFigure 1. Distribution of Wait Times Across Site Types for Pediatric Viral and Respiratory Visits, January 1, 2021, to December 31, 2022

eFigure 2. Distribution of Lengths of Stay Across Site Types for Pediatric Viral and Respiratory Visits, January 1, 2021, to December 31, 2022

This supplemental material has been provided by the authors to give readers additional information about their work.

© 2023 Janke AT et al. *JAMA Network Open*

eTable 1. Most Common Chief Complaints Defining Sample Cohort

**Description**	**N**	**%**
**Fever**	56,933	18.9%
**Cough**	39,457	13.1%
**Shortness of breath**	21,960	7.3%
**Vomiting**	16,827	5.6%
**Sore Throat**	7,067	2.3%
**Respiratory Distress**	6,701	2.2%
**Nasal Congestion**	4,061	1.3%
**Ear Pain**	3,957	1.3%
**Chest Pain**	1,843	0.6%
**Infection Screening**	1,436	0.4%

Table depicts the most common free text chief complaints that were used to define the cohort of pediatric viral and respiratory ED visits studied in the analysis.

© 2023 Janke AT et al. *JAMA Network Open*

eTable 2. Most Common Diagnosis Codes Defining Sample Cohort

Description	N	%
Acute upper respiratory infection, unspecified	40,181	13.3%
Fever, unspecified	24,011	8.0%
Coronavirus disease-2019 (COVID-19)	13,985	4.6%
Viral infection, unspecified	11,152	3.7%
Acute obstructive laryngitis [croup]	10,537	3.5%
Influenza with other respiratory manifestations	7,260	2.4%
Acute bronchiolitis, unspecified	5,986	2.0%
Nausea with vomiting, unspecified	5,960	2.0%
Acute bronchiolitis due to respiratory syncytial virus	5,809	1.9%
Unspecified asthma with (acute) exacerbation	5,118	1.7%

Table depicts the most common diagnosis code descriptions that were used to define the cohort of pediatric viral and respiratory ED visits studied in the analysis

© 2023 Janke AT et al. *JAMA Network Open*

eTable 3. Sample Emergency Department Sites from the Michigan Emergency Department Improvement Collaborative

Site Category	Pediatric Visits (% Overall Visits)	Admissions (% Pediatric Visits)	PICU Site	Teaching Site (ACGME)	Urbanicity Designation
**Children's Hospitals**					
1	99.7%	9.4%	Y	Y	1
2	98.8%	11.5%	Y	Y	1
3	30.3%	18.2%	Y	Y	1
**Urban, Pediatric Volume >10%**					
1	18.7%	N/A	Y	N	1
2	18.4%	14.3%	N	Y	1
3	16.7%	2.7%	N	N	1
4	15.4%	N/A	N	Y	1
5	15.2%	2.1%	N	Y	1
6	14.6%	2.7%	N	Y	1
7	13.9%	1.9%	N	Y	1
8	12.0%	N/A	N	Y	1
9	10.4%	4.0%	N	Y	2
**Urban, Pediatric Volume <10%**					
1	8.7%	4.0%	N	Y	1
2	8.6%	3.6%	N	Y	1
3	7.5%	N/A	N	Y	1
4	7.1%	N/A	N	Y	1
5	7.0%	1.8%	N	Y	1
6	4.0%	1.6%	N	Y	1
7	2.2%	10.0%	N	Y	1
**Rural ED**					
1	24.5%	N/A	N	N	10
2	20.9%	N/A	N	N	10
3	20.8%	N/A	N	N	7
4	18.9%	N/A	N	Y	4
5	15.9%	N/A	N	N	7
6	10.9%	10.4%	N	Y	4

Teaching status is reported according to the presence of Accreditation Counsel on Graduate Medical Education (ACGME) program present at the site. *Urbanicity is defined according to Rural-Urban Commuting Area (RUCA) codes as in prior research on U.S. ED locations; 1=Metropolitan area core, 2=Metropolitan area high commuting area, 4=Micropolitan area, 7=Small town, 10=Rural area.

© 2023 Janke AT et al. *JAMA Network Open*

eTable 4. Sample Sociodemographic Characteristics of Pediatric Viral and Respiratory Visits

	**Children's Hospital (N=3)**	**Urban, Pediatric Volume >10% (N=9)**	**Urban, Pediatric Volume <10% (N=7)**	**Rural ED (N=6)**	**P-Values**
**Age**					<0.001
< 4 weeks	2,576 (1.4%)	846 (1.2%)	191 (1%)	204 (1%)	
4 week – 3 months	7,367 (3.9%)	2,380 (3.3%)	433 (2.2%)	588 (2.9%)	
4 months – 23 months	56,918 (29.9%)	18,979 (26.7%)	4,259 (21.6%)	5,286 (25.8%)	
2 – 4 years	48,714 (25.6%)	16,296 (22.9%)	4,222 (21.4%)	4,989 (24.3%)	
5 – 11 years	48,135 (25.3%)	18,230 (25.6%)	5,467 (27.7%)	5,380 (26.2%)	
12-17 years	26,606 (14%)	14,408 (20.3%)	5,133 (26%)	4,081 (19.9%)	
**Sex**					<0.001
Male	101,267 (53.2%)	36,149 (50.8%)	10,071 (51.1%)	10,533 (51.3%)	
Female	89,049 (46.8%)	34,990 (49.2%)	9,634 (48.9%)	9,995 (48.7%)	
**Race Category**					<0.001
American Indian/Alaskan Native	312 (0.2%)	320 (0.4%)	196 (1%)	210 (1%)	
Asian	1,619 (0.9%)	903 (1.3%)	514 (2.6%)	87 (0.4%)	
Black/African American	70,102 (36.8%)	15,078 (21.2%)	7,553 (38.3%)	433 (2.1%)	
More Than One Race	4,599 (2.4%)	1,913 (2.7%)	159 (0.8%)	475 (2.3%)	
Native Hawaiian/Pacific Islander	110 (0.1%)	168 (0.2%)	60 (0.3%)	36 (0.2%)	
Some Other Race	44,597 (23.4%)	8,759 (12.3%)	3,197 (16.2%)	2,821 (13.7%)	
White	53,958 (28.4%)	43,679 (61.4%)	8,018 (40.7%)	16,280 (79.3%)	
**Ethnicity Category**					<0.001
Hispanic/Latino	17,743 (9.3%)	8,124 (11.4%)	2,070 (10.5%)	1,317 (6.4%)	
Not Hispanic/Latino	171,692 (90.2%)	63,015 (88.6%)	17,635 (89.5%)	19,174 (93.4%)	
**Insurance Status**					<0.001
Medicaid	49,242 (25.9%)	40,874 (57.5%)	12,036 (61.1%)	219 (1.1%)	
Medicare	92 (0%)	92 (0.1%)	65 (0.3%)	<10	
Private	131,646 (69.1%)	26,957 (37.8%)	7,067 (35.9%)	19941 (97.1%)	
Self-Pay	5,304 (2.8%)	264 (0.4%)	153 (0.8%)	127 (0.6%)	
Other/Missing	4,032 (2.1%)	2,952 (4.1%)	384 (1.9%)	241 (1.2%)	

Sample includes 25 EDs from the Michigan Emergency Department Improvement Collaborative. This includes ED visits for children ages 0-18 years with a chief complaint or diagnosis code for a viral respiratory condition. Chi-square test p-values are reported for differences across groups.

© 2023 Janke AT et al. *JAMA Network Open*

eTable 5. Sample Pediatric Viral Respiratory Visit Characteristics

	Children's Hospital (N=3)	Urban, Pediatric Volume >10% (N=9)	Urban, Pediatric Volume <10% (N=7)	Rural ED (N=6)	P-Values
**Common Primary Diagnoses**					<0.001
URI Sign/Symptom	37,524 (19.7%)	16,229 (22.8%)	4,725 (24%)	5,188 (25.3%)	
Asthma	11,262 (5.9%)	2,401 (3.4%)	522 (2.6%)	414 (2%)	
Bronchiolitis	8,579 (4.5%)	2,748 (3.9%)	290 (1.5%)	871 (4.2%)	
COVID	10,468 (5.5%)	4,544 (6.4%)	1547 (7.9%)	1,494 (7.3%)	
Croup	6,849 (3.6%)	2,242 (3.2%)	486 (2.5%)	960 (4.7%)	
Fever, unspecified	15,641 (8.2%)	5,951 (8.4%)	1202 (6.1%)	1,217 (5.9%)	
Influenza	4,950 (2.6%)	2,371 (3.3%)	773 (3.9%)	840 (4.1%)	
Pneumonia	2,307 (1.2%)	697 (1%)	143 (0.7%)	231 (1.1%)	
Respiratory Failure	1,031 (0.5%)	240 (0.3%)	29 (0.1%)	57 (0.3%)	
Other	91,705 (48.2%)	33,716 (47.4%)	9,988 (50.7%)	9,256 (45.1%)	
**Acuity**					<0.001
1	3,246 (1.7%)	454 (0.6%)	214 (1.1%)	143 (0.7%)	
2	37,337 (19.6%)	9,297 (13.1%)	3,261 (16.5%)	2,578 (12.6%)	
3	64,426 (33.9%)	31,737 (44.6%)	9,752 (49.5%)	6,628 (32.3%)	
4	79,032 (41.5%)	26,065 (36.6%)	5,996 (30.4%)	9,676 (47.1%)	
5	5,227 (2.7%)	3,044 (4.3%)	356 (1.8%)	1,264 (6.2%)	
**Complex Chronic Conditions**					<0.001
0	175,885 (92.4%)	70,197 (98.7%)	19,530 (99.1%)	20,164 (98.2%)	
1	8,843 (4.6%)	778 (1.1%)	165 (0.8%)	272 (1.3%)	
2+	5,588 (2.9%)	164 (0.2%)	10 (0.1%)	92 (0.4%)	
**Disposition**					<0.001
Discharge	154,823 (81.4%)	62,158 (87.4%)	16,697 (84.7%)	18,478 (90%)	
Admit	32,947 (17.3%)	4,324 (6.1%)	670 (3.4%)	811 (4%)	
Transfer	192 (0.1%)	2,529 (3.6%)	1,206 (6.1%)	574 (2.8%)	
**Outcomes**					<0.001
Wait Time >4 Hours	6,342 (3.3%)	223 (0.3%)	337 (1.7%)	205 (1.0%)	
Lengths of Stay >12 Hours	10,120 (5.3%)	1,615 (2.3%)	596 (3.0%)	414 (2.0%)	
ED Revisit within 72 Hours	7,066 (3.7%)	2,537 (3.6%)	356 (1.8%)	901 (4.4%)	

Sample includes 25 EDs from the Michigan Emergency Department Improvement Collaborative. This includes ED visits for children ages 0-18 years with a chief complaint or diagnosis code for a viral respiratory condition. Chi-square test p-values are reported for differences across groups.

© 2023 Janke AT et al. *JAMA Network Open*

eTable 6. High Acuity and Transfer Visits for Lengths of Stay >12 Hours During the Surge Period

	Total LOS >12 Hours (n)	High-Acuity LOS>12 Hours (n, %)	Transfers LOS >12 Hours (n, %)
Children's Hospitals (n=3)	3,789	2,331 (61.5%)	32 (0.8%)
Urban, Pediatric >10% (n=9)	425	245 (57.6%)	123 (28.9%)
Urban, Pediatric <10% (n=7)	133	104 (78.2%)	74 (55.6%)
Rural ED (n=6)	176	118 (67.0%)	42 (23.9%)

Total ED visits with lengths of stay (LOS) >12 hours, high-acuity visits with LOS >12 hours, and ED visits resulting in transfer with LOS >12 hours are reported for the surge period (September 1 through December 31, 2022). High-acuity visits are defined by those with an ESI of 1 or 2.

eTable 7. High-Acuity and Hospitalizations for ED Revisits During the Surge Period

	Total Revisits (n)	High-Acuity Revisits (n, %)	Hospitalized Revisits (n, %)
Children's Hospitals (n=3)	1,842	510 (27.7%)	483 (26.2%)
Urban, Pediatric >10% (n=9)	891	195 (21.9%)	215 (24.1%)
Urban, Pediatric <10% (n=7)	115	28 (24.3%)	25 (21.7%)
Rural ED (n=6)	269	31 (11.5%)	26 (9.7%)

Total ED revisit counts, high-acuity revisits, and hospitalized revisits are reported for the surge period (September 1 through December 31, 2022). ED revisits are those with a recent preceding discharged visits within 72 hours. High-acuity revisits are defined by those with an ESI of 1 or 2. Hospitalized revisits are those with a disposition for hospital admission or transfer to another acute care facility.

© 2023 Janke AT et al. *JAMA Network Open*

eFigure 1. Distribution of Wait Times Across Site Types for Pediatric Viral and Respiratory Visits, January 1, 2021, to December 31, 2022

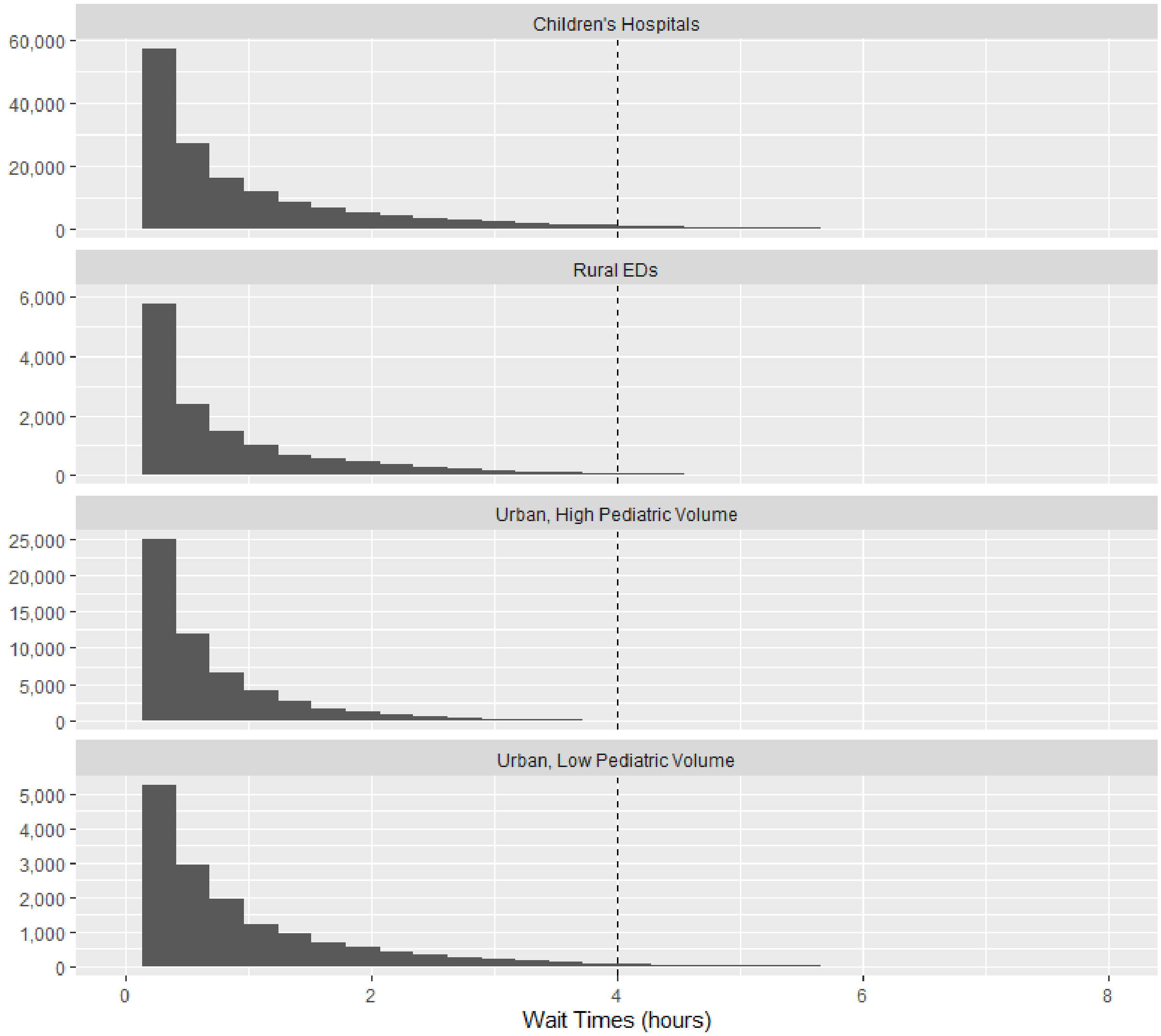

Caption: Figure depicts distribution of wait times by hour across site types for pediatric viral and respiratory ED visits in the analyses. Dashed vertical line depicts 4-hour cutoff where wait time outcome was dichotomized.

© 2023 Janke AT et al. *JAMA Network Open*

eFigure 2. Distribution of Lengths of Stay Across Site Types for Pediatric Viral and Respiratory Visits, January 1, 2021, to December 31, 2022

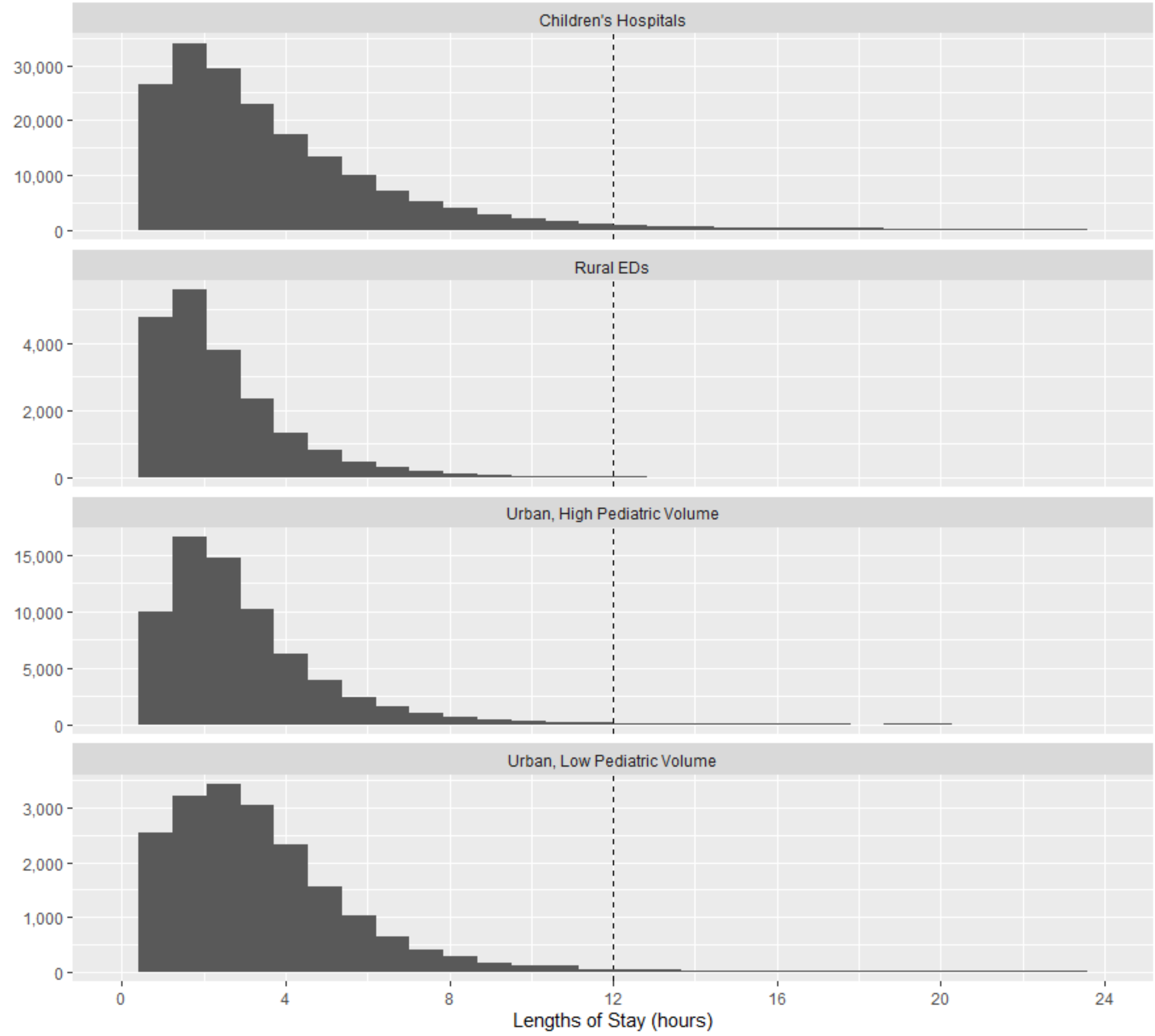

Caption: Figure depicts distribution of lengths of stay by hour across site types for pediatric viral and respiratory ED visits in the analyses. Dashed vertical line depicts 12-hour cutoff where length of stay outcome was dichotomized.

© 2023 Janke AT et al. *JAMA Network Open*
