# Supplementary material for: Emergency Department Care for Children During the 2022 Viral Respiratory Illness Surge
Source: JAMA Netw Open. 2023 Dec 7;6(12):e2346769. doi: 10.1001/jamanetworkopen.2023.46769 (PMC10704279; doi:10.1001/jamanetworkopen.2023.46769)
Supplement: Supplement 2. — Data Sharing Statement [file jamanetwopen-e2346769-s002.pdf]

Data Sharing Statement

Janke. Emergency Department Care for Children During the 2022 Viral Respiratory Illness Surge. *JAMA Netw Open*. Published December 08, 2023.
doi:10.1001/jamanetworkopen.2023.46769

Data
Data available: No
